# *In vitro* homology search array comprehensively reveals highly conserved genes and their functional characteristics in non-sequenced species

**DOI:** 10.1186/1471-2164-11-S4-S9

**Published:** 2010-12-02

**Authors:** Atsushi Ogura, Masa-aki Yoshida, Mutsumi Fukuzaki, Jun Sese

**Affiliations:** 1 Ochadai Academic Production, Ochanomizu University, Ohtsuka 2-1-1, Bunkyo, Tokyo, Japan; 2 Department of Computer Science, Ochanomizu University, Ohtsuka 2-1-1, Bunkyo, Tokyo, Japan

## Abstract

**Background:**

With the increase in genomic and transcriptomic data produced by the recent advancements in next generation sequencers and microarrays, it is now easier than ever to conduct large-scale comparative genomic studies for familiar species. However, there are more than ten million species on earth, and the study of all remaining species is not realistic in terms of cost and time. There have been a number of attempts at using microarrays for cross-species hybridization; however, those approaches only utilized the same probes for each species or different probes designed from orthologous genes. To establish easier and cheaper methods for the large-scale comparative genomic study of non-sequenced species, we developed an *in vitro* homology search array with the aid of a bioinformatic approach to probe design.

**Results:**

To perform large-scale genomic comparisons of non-sequenced species, we chose squid, one of the most intelligent species among Protostomes, for comparison with human genes. We designed a microarray using human single copy genes and conducted microarray experiments with mRNAs extracted from the squid. Multi-copy genes could not be detected using the microarray in this study because their sequence similarity caused cross-hybridization. A search for squid homologous genes among human genes revealed that 68% of the human probes tested showed the expression of squid homolog genes and 95 genes were confirmed to be expressed highly in squid. Functional classification analysis showed that these highly expressed genes comprise DNA binding proteins, which are under pressure of DNA level mutation and, consequently, show high similarity at the nucleotide level.

**Conclusions:**

Our array could detect homologous genes in squids and humans in spite of the distant phylogenic relationships between the species. This experimental method will be useful for identifying homologs in non-sequenced species, for the development of genetic resources and for the collection of information on biodiversity, particularly when using the genome of sibling or closely related species.

## Background

The recent development of next-generation sequencers has allowed us to sequence the complete genome of various species easily and rapidly [1,2]. Even though deep sequencing is the fastest and cheapest method to date, the species examined by deep sequencing are still limited to model organisms and species that are medically or commercially important. For example, 36 complete genomes are available among mammals, which occupy only 0.3% of species on the earth, whereas only 16 genomes including 10 fruit-fly genomes are available for insect genome, which comprise more than 50% of all species [3-6]. From the viewpoint of biodiversity, we need to know the genomes of as wide a range of species as possible to allow for environmental protection, to provide material for diversified genetic resources and to promote the basic sciences such as ecology, genetics and evolution [7-9]. For species not currently included in genome projects, it is still possible to determine genes and their sequences by constructing cDNA libraries and cloning with RACE methods. Large-scale genomic studies to better understand biological diversity, and evolutionary systems and mechanisms, however, are not possible via these strategies because they are limited to the use of only a few samples. On the other hand, with the spread of next-generation sequencers across the globe, there has been a rapid increase in the accumulation of DNA sequence data [10], which makes it difficult to undertake traditional bioinformatic analyses such as homology searches. Thus, there is a need to develop new methods for large-scale genomic studies of non-sequenced species. Our aim is not to find all homologous genes between sequences, which is not possible in case where RNA is absent or weak. Indeed, detection of all homologous genes is not possible using microarray methods as such experimental methods tend to result in false positive and true negative estimations. There have been several attempts to examine gene expression profiles using microarray [11-20], but the challenge to search homologs themselves by microarray is unique and novel.

Toward this end, we have developed a novel strategy to pursue large-scale genomic studies using a microarray. As a first step, we tried to identify homologous sequences between species diverged hundreds of millions of years ago. In this study, we selected humans and squids, for a comparison of mammals and cephalopods. We choose these species because though they diverged in the pre-Cambrian period and evolved independently, both acquired elaborate eyes and brains that are remarkable among the two major classes of Bilateral animals; i.e., Deuterostomes and Protostomes [21]. Accordingly, a comparison of genes and gene sets between these species is of particular interest for the understanding of animal evolution. There is no need to conduct *in vitro* homology searches if both genomes are sequenced, so we have chosen squid as a non-sequenced species. There are, of course, many candidates for such a study; for example, humans vs mice or flies vs mosquitoes, but we sought to test our study in non-sequenced species from scratch, and assess the sensitivity for homology detection between relatively distant species. We will continue work on other pairs once we have obtained reliable results for humans and squids.

## Results

### Can an *in vitro* homology search array detect homologs in remotely related species?

**Figure 1:**
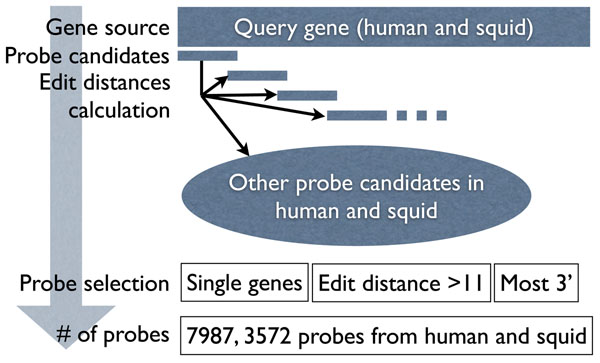
**Procedure of probe design for *in vitro* homology search array.** For every genes stored in the public database for human and squid genes, we extracted 60 bp candidate probes at intervals of 50bp. Edit distances were calculated and the appropriate probes were selected according to the following criteria; single genes, more than 11 edit distance, most 3' probe. A total of 11,559 probes were designed on the array, including 7,987 human probes and 3,572 squid probes.

To search for homologies between human and pygmy squid genes, we developed a novel *in vitro* homology search array consisting of probes for human and squid genes. The total number of probes on the array was 11,559 including 7,987 probes designed from human genes and 3,572 probes designed from pygmy squid genes (Figure 1). To avoid cross hybridization against very similar probes on the same array, we have selected appropriate region in the coding genes in both species (see Method).

**Figure 2:**
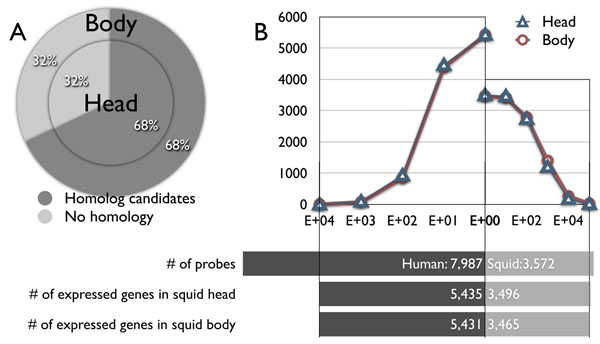
**Microarray experiments.** Two independent microarray experiments were performed using pygmy squid head mRNAs and pygmy squid body mRNA using the same microarray design. A) Out of 7,987 human probes, 5,435 and 5,431 probes could detect significant expression of squid genes in head and body, that stands for 68 % of human probes. B) Distribution of expression intensities were shown. Left side graph represents the expression intensities of squid head and body mRNAs detected by human probes. Y-axis represents the cumulative total number of probes, and X-axis represents each expression intensities by log-scale. Right side graph represents the expression intensities of squid head and body mRNAs detected by squid probes.

Next, we performed microarray experiments using pygmy squid mRNA. The mRNAs were obtained from the whole embryonic head (head), including the brain and eye, and the remaining portion of the embryo (body) for two purposes; to conduct two independent microarray experiments to test for reliability and to observe the changes in gene expression between different tissues. We found the significant expression of 5,435 and 5,431 probes out of 7,987 human probes in the head and the body of pygmy squids, respectively, using Agilent microarray scanner protocols (Figure 2). This indicates that about 68 % of the human probes tested were homologous genes (Figure 2).

### Are these genes really homologs in pygmy squids?

It is possible that the number of expressed genes is over-estimated due to hybridization to non-target genes on the microarray even though we used standard scanner protocols and our probes were carefully designed to avoid hybridization to non-target genes. Note that such cross-hybridizations will dramatically reduce expression intensities. We first checked the number of probes for which the intensities were high among human probes and found that the expression levels (probe intensities) of most of these genes were less than 1,000 (1E+03 ≒ 10bit) expression intensities on a 20bit dynamic range as measured by an Agilent microarray scanner. This result implies that most probes detected non-target genes.

**Figure 3:**
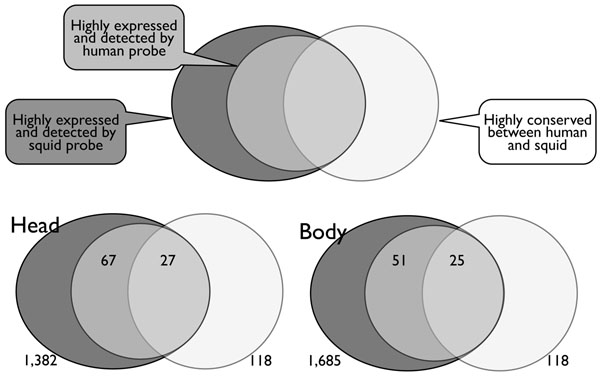
**Highly expressed genes tend to be conserved rather than other genes.** Venn diagrams show the relationships among highly expressed genes detected by human probes and squid probes, and highly conserved genes calculated by blastn search.

To dismiss this possibility, we carefully checked probes with high intensities. We set the threshold as 1,000 expression intensities and extracted the highly expressed genes having intensities meeting this criterion as potential homologous genes. We then determined 94 and 76 homologs in samples of head and body respectively. Out of the 94 and 76 genes, 27 and 25 genes are already known in the public databases; thus, we can conclude that highly expressed genes in our array are homologous genes. Furthermore, 67 and 51 genes are newly identified homologs (Figure 3, Additional Files 1 and 2, based on probe sequence data in Additional file 3).

We then validated whether or not our *in vitro* homology search array can identify genes already known to be highly conserved between humans and squids. We have checked the expressed genes in squid samples using 3,572 squid probes on the array, and found 1,382 and 1,685 out of 3,572 genes are expressed at intensities higher than the threshold, in head and body respectively. A comparison of squid ESTs from the GenBank against human genes designed on our array showed that 118 genes have a less than 1e-04 e-value in blastn search against human genes, and these were then used for the validation of homologies (Figure 3). Out of 118 homologous genes, 27 and 25 genes were detected on our array in head and body, which is equivalent to the average detection coverage of all probes and indicates that our strategy is a useful method for the detection of homologous genes in a query sample (Figure 3) and could successfully detect homologous genes on our array.

### What kinds of genes have been identified in this in vitro homology search?

Not only does our array estimate homologous, but we can also detect expression discrepancies between two different samples; i.e., the squid head and body. It is possible that sequence identity of squid genes against human probes might affect signal intensity in the microarray. However, the same probe with a different signal intensity provides reliable information on changes in gene expression. We identified 15 genes in which expression varied significantly between samples for the squid head and body (Table 1). All 15 genes were newly identified by our method.

**Table 1:** The list of differential expressed genes between head and body of pygmy squid

ProbeName	Fold change (Head / Body)	Regulation (Head / Body)	Annotation in Human	Newly identified in Squid
A_23_P145841_923	1062.9779	down	sclerostin domain containing 1 (SOSTDC1)	O
A_23_P150457_1809	192.46806	down	lymphatic vessel endothelial hyaluronan receptor 1 (LYVE1)	O
A_23_P47616_2403	189.59702	down	folate hydrolase 1 (FOLH1)	O
A_24_P3 19715_ 1231	157.6817	down	disulfide isomerase family A, member 6 (PDIA6)	O
A_23_P214529_3619	144.43817	down	zinc finger protein 192 (ZNF192)	O
A_23_P57401_3729	109.048706	down	leucine-zipper-like transcription regulator 1 (LZTR1)	O
A_23_P143526_508	99.44422	down	S100 calcium binding protein B (S100B)	O
A_23_P78903_1083	92.95913	down	cyclin N-terminal domain containing 2 (CNTD2)	O
A_23_P5415_1270	71.51518	up	NIF3NGG1 interacting factor 3-like 1 (NIF3L1)	O
A_24_P319989_1071	57.572376	up	caveolin 3 (CAV3)	O
A_24_P71244_4544	56.09429	up	phosphoinositide-3-kinase, catalytic, delta polypeptide (PIK3CD)	O
A_23_P116902_1260	51.13099	up	ADP-ribosyltransferase 4 (ART4)	O
A_23_P31536_420	50.551243	down	single-stranded DNA binding protein 1 (SSBP1)	O
A_23_P44257_1197	45.264664	up	COMM domain containing 8 (COMMD8)	O
A_23_P205265_1700	45.247013	down	eukaryotic translation initiation factor 5 (EIF5)	O

Statistically significant differential expressions were extracted, and more than 40 fold change were listed. Sequences of ProbeName can be obtained from Additional file 3. containing probe information.

**Figure 4:**
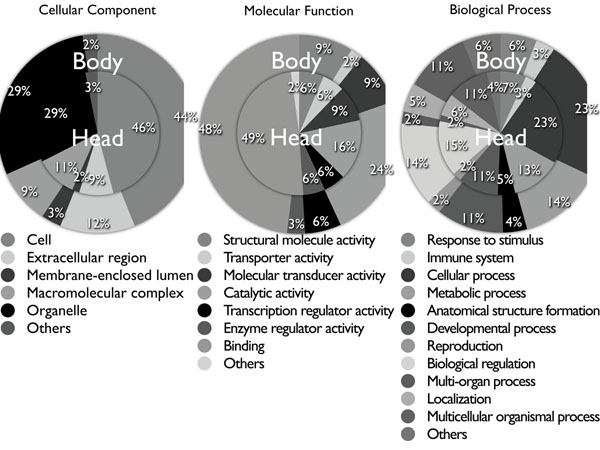
**GO classification of homologs identified by *in vitro* homology search array**. GO classification of genes expressed in head and body of pygmy squid. Blast2GO software was used in this study, and showed the three different categories at the level 2 of Cellular Component, Molecular Function, and Biological Process.

The list of homologous genes indicates that 84% of the genes are function-known in human genome annotation, and GO classification analysis [22,23] suggests that the majorities of the genes are DNA-binding proteins (GO: Molecular Function) or are involved in cellular process and regulation (GO: Biological Process) (Figure 4). This seems to be a reasonable result because, even more than 500 million years after the divergence between humans and squids, DNA binding proteins are usually included among highly conserved genes due to selection pressure [24-27]. GO functional composition between head and body was not so different, mainly because these genes are highly conserved between distant species and showed essential gene set conserved at nucleotide level.

## Discussion

### Novel approaches to the estimation of homologous genes in non-sequenced species

Our *in vitro* homology search array was able to estimate more than five thousand homologous genes in squid and human, which represents 68% of the human genes tested in this study. This indicates that a large-scale genomic comparative homology search is suitable for non-sequenced species. We were able to obtain this result from a single experiment, and by doing further experiments, we expect the number homologous genes identified to increase. As only 85 nucleotides and 9,079 ESTs for pygmy squid have been submitted to GenBank as of May 2010, our approach is a very efficient technique by which to identify homologous genes. Our method will be useful particularly for sibling genome-sequenced species.

Several cross-species microarrays have been designed for various purposes, and a number of papers have provided thoughtful insights into the design and analysis of microarray experiments [11-13]. Other approaches have focused on the genomic and transcriptomic diversity within mammals or primates [14,15]. Specific targets, such as nitrogen availability and toxicogenomics, could be pursued through the use of cross-species arrays [16,17]. Heterologous or duplicated regions are hard target to detect with microarrays because of cross hybridization; however, careful assessment and analysis of those data have been reported recently [18,19]. Unlike these previous studies, our approach has a specialized target, the identification of homologous genes between distant species.

There is a vogue strategy for the large-scale genomics based on next-generation sequencers[20], but the advantage of our method lies in the low experimental costs. Microarray-based experiments are 10 to 20 times cheaper than current next-generation sequencing methods, and this reason alone is sufficient to encourage the application of this method to millions of species to promote the the study of biodiversity. *In silico* homology search methods, including normal homology searches such as blast, are, on the contrary, powerful tools with which to detect both close and distant homologies, but they require query sequences that are usually only partially available in non-sequenced species.

### Limitations and solutions

There are some difficulties in detecting homologous sequences by experimental methods. First, in comparing amino acid sequences, DNA sequences are easily mutated over long periods. We often use homology searches (nucleotide-nucleotide searches) for closely related species, and translated searches of amino acid databases for distantly related species. As it is not possible to perform translated searches in experiments, we can only detect homologous regions if their sequences are well conserved and protected from mutations by slow molecular clocks. We are now assessing the efficiency of hybridization by conducting artificial cross-hybridization microarray experiments (in preparation).

Second, we need to focus on gene coding regions for homology detection because it is likely that intergenic regions are too highly mutated for experimental detection. We, therefore, used mRNAs to search for homologous genes between two different species. In the case of mRNAs, problems still arise in the distribution of gene expression intensities.

Third, probe design in the microarray is also problematic. If probes with similar sequences are used in the same microarray, it is possible to detect expressed genes with two different probes as cross hybridization. To avoid this problem, we have calculated probe sequences in which edit distances are at least 70% different from the most similar probes. We are currently investigating the effect of cross hybridization by designing two different microarrays with a few artificial mutation sites (in preparation). The potential for cross hybridization should be considered carefully when designing microarrays.

Fourth, there is a problem with duplicated genes and gene families. These genes have hundreds of homologous siblings that prevent experimental homology searches due to cross hybridization. To prevent estimating false positive homologs, we have removed duplicated genes from the array. Our method was aimed at detecting homologous genes. The search for homology between species proceeds more efficiently if we focus on single copy genes. Detection of homologs of multi-copy genes would require a different probe design strategy.

### Possible applications to other species such as more closely related species

We have tested this methods on humans and squid, which are distant from each other in terms of nucleotide sequence conservations. We could still identified 95 homologous genes, and estimated more than 5,400 candidate genes in squid that could have homologs in humans. This indicates that if we apply this method with more probes from humans, we may identify an increased number of homologous genes in squid. This method may also be applied to other species groups such Primates, Rodents, or Diptera (flies), thus allowing larger scale and cheaper genomic comparisons.

## Conclusions

We have developed an *in vitro* homology search array for the estimation of distantly related homologs. It allows the estimation of homologous genes between humans and squids, which diverged more than 500 million years ago. Some genes are, of course, false positive estimations, but the highly expressed genes are thought to be homologous genes. This experimental strategy will be particularly valuable when the explosion of DNA sequences data expected to be produced by next-generation sequencers leads to computational limits on the performance of homology searches.

## Methods

### Collection of the pygmy squid

Japanese pygmy squid, *Idiosepius paradoxus* (Ortmann, 1881) were captured in the shallow waters along the southern coast of Chita Peninsula in central Honshu, Japan and maintained in a tank at the Ochanomizu University. Spawned egg masses were transferred to a Petri dish and kept at 20°C. To determine the developmental stages of the embryo, the criteria established by Yamamoto (1988) were used [24].

### mRNA extraction

Total RNA extractions were performed by using E.Z.N.A.® Mollusc RNAKit (Omega Bio-Tek Inc., Norcross, GA, USA.) following the manufacturer's instructions. Total RNA samples were extracted from the embryonic head part and the remaining portion of the body of pygmy squid at stage-25. The embryonic head (including eyes and optic lobe) was cut using forceps and collected separately from the remaining body and used for total RNA extraction.

### *in vitro* homology search array

We generated microarray probes for squids, *I*. *paradoxus,* using the following procedure. First, we extracted 60 bp candidate probes at intervals of 50bp from each squid genes because the probe length for the Agilent microarray is 60bp. Second, for each candidate probe, we calculated the minimum edit distance between the probe and the all squid genes except the gene containing the candidate probe. With this edit distance, we can avoid cross hybridization between probe and non-target genes. A small distance between the probe and the genes indicates a high possibility of hybridization between them. If the distance was more than eleven and the probe located more than 20bp away from the 3'-end of the gene to which the probe belonged, we regarded the probe as appropriate. We designed the microarray using these probes that meet these criteria. When multiple appropriate probes could be selected from a single gene, we selected the probe closest to the 3'-end of the gene.

### Human and squid probe design

As probe sequences for human genes, we utilized original sequences provided by Agilent Technologies. However, the sequences are designed using UCSC Human Genome build Hg 18, which is a slightly out of date human genome assembly. Hence we chose probes whose sequences exist in a single gene on UCSC Human Genome build Hg 19. For squid genes, we collected available EST sequences from Genbank and also designed their probes in the same procedure. To remove the similar probes among the human and squids probes, we furthermore selected probes whose minimum edit distances for *I*. *paradoxus* genes were more than eleven. Duplicated genes or multi-copy genes were removed during this protocol. By this procedure, we finally selected 7,937 and 3,572 probes from human and squid respectively.

### Microarray experiments and analysis

To perform the comparative analysis between humans and squid, we applied total RNA samples from humans and the pygmy squid onto the custom Agilent microarray 8x15K format. The microarray comprises 11,559 target sequences selected by the process mentioned above. Total RNA from the embryonic head of the pygmy squid and the remaining body portion were labeled and applied separately to the array analysis. Briefly, 0.5 µg of total RNA from each samples was used to synthesize fluorescent-labelled cRNA using Cyanine 3-CTP (CY3c) as described in the manual (Agilent Quick Amp Labeling Kit, one-color). Labeled DNA was hybridized for 16hr at 65°C on the custom array. After hybridization the microarray slides were washed using the standard protocol (Agilent Technologies, USA) and scanned on an Agilent microarray scanner. Data were analyzed using the Agilent Feature Extraction Software (v10.7). The software normalize for any differences in dye signal intensity, then, calculates a reliable log ratio, p-value, and log ratio error for each feature to give a confidence measure in the measured log ratio. Microarray data was submitted to CIBEX.

## Authors' contributions

AO and JS conceived and designed the study. MY performed mRNA extraction and conducted microarray analyses. MF applied the bioinformatics approach to design the array. AO, MF and JS conducted the microarray experiments. AO, MY and JS wrote the paper. All authors discussed the results and commented on the manuscript.

## Competing interests

The authors declare that they have no competing interests.

## Supplementary Material

Additional file 1 These 67 new homologs were selected because they are detected with high intensities (>1000) in the array. Click here for file

Additional file 2 These 51 new homologs were selected because they are detected with high intensities (>1000) in the array. Click here for file

Additional file 3 Probe Name and Probe sequence were shown in the probe sequence file. Click here for file

## References

[B1] Margulies Genome sequencing in microfabricated high-density picolitre reactors Nature 2005 437 7057 376 80 16056220 10.1038/nature03959 PMC1464427

[B2] Stephen Large-scale appearance of ultraconserved elements in tetrapod genomes and slowdown of the molecular clock Mol Biol Evol 2008 25 2 402 8 10.1093/molbev/msm268 18056681

[B3] Erwin TL Tropical forests: their richness in Coleoptera and other arthropod species The Coleopterists Bulletin 1982 36 74 75

[B4] Novotny Low host specificity of herbivorous insects in a tropical forest Nature 2002 416 841 844 10.1038/416841a 11976681

[B5] Wheeler QD Ontogeny and character phylogeny Cladistics 1990 6 225 264 10.1111/j.1096-0031.1990.tb00542.x

[B6] Liolios K Chen IM Mavromatis K Tavernarakis N Hugenholtz P Markowitz VM Kyrpides NC The Genomes On Line Database (GOLD) in 2009: status of genomic and metagenomic projects and their associated metadata NAR Epub 2009 Nov 13 10.1093/nar/gkp848 PMC2808860 19914934

[B7] Turnbaugh The human microbiome project Nature 2007 449 7164 804 10 10.1038/nature06244 17943116 PMC3709439

[B8] Petrosino Metagenomic pyrosequencing and microbial identification Clin Chem 2009 55 5 856 66 10.1373/clinchem.2008.107565 19264858 PMC2892905

[B9] Doebeli Ispolatov Complexity and diversity Science 2010 328 5977 494 7 10.1126/science.1187468 20413499

[B10] Bhatia Dealing with database explosion: a cautionary note Science 1997 276 5319 1724 5 10.1126/science.276.5319.1724 9206831

[B11] Pariset Microarrays and high-throughput transcriptomic analysis in species with incomplete availability of genomic sequences N Biotechnol 2009 25 5 272 9 10.1016/j.nbt.2009.03.013 19446516

[B12] Kuhn Cross-species and cross-platform gene expression studies with the Bioconductor-compliant R package 'annotationTools' BMC bioinformatics 2008 9 26 10.1186/1471-2105-9-26 18201381 PMC2267709

[B13] Lu Cross species analysis of microarray expression data Bioinformatics 2009 25 12 1476 83 10.1093/bioinformatics/btp247 19357096 PMC2732912

[B14] Adjaye Cross-species hybridisation of human and bovine orthologous genes on high density cDNA microarrays BMC Genomics 2004 5 1 83 10.1186/1471-2164-5-83 15511299 PMC535340

[B15] Oshlack Using DNA microarrays to study gene expression in closely related species Bioinformatics (Oxford, England) 2007 23 10 1235 42 10.1093/bioinformatics/btm111 17384014

[B16] Schönig Cross-species hybridization with Fusarium verticillioides microarrays reveals new insights into Fusarium fujikuroi nitrogen regulation and the role of AreA and NMR Eukaryotic Cell 2008 7 10 1831 46 10.1128/EC.00130-08 18689524 PMC2568065

[B17] Calley Microarray probe mapping and annotation in cross-species comparative toxicogenomics Methods Mol Biol 2008 460 159 83 18449487 10.1007/978-1-60327-048-9_8

[B18] Degletagne Transcriptome analysis in non-model species: a new method for the analysis of heterologous hybridization on microarrays BMC Genomics 2010 11 1 344 10.1186/1471-2164-11-344 20509979 PMC2901317

[B19] Machado Renn A critical assessment of cross-species detection of gene duplicates using comparative genomic hybridization BMC Genomics 2010 11 1 304 10.1186/1471-2164-11-304 20465839 PMC2876127

[B20] Bellin Combining next-generation pyrosequencing with microarray for large scale expression analysis in non-model species BMC Genomics 2009 10 1 555 10.1186/1471-2164-10-555 19930683 PMC2790472

[B21] Ogura Comparative analysis of gene expression for convergent evolution of camera eye between octopus and human Genome Res 2004 14 8 1555 61 10.1101/gr.2268104 15289475 PMC509264

[B22] Stefan Götz Juan García-Gómez Miguel Javier Terol Tim Williams D. María Nueda José Montserrat Robles Manuel Talón Joaquín Dopazo Ana Conesa High-throughput functional annotation and data mining with the Blast2GO suite Nucleic Acids Res 2008 36 10 3420 3435 June 10.1093/nar/gkn176 18445632 PMC2425479

[B23] Ana Conesa Stefan Götz Juan García-Gómez Miguel Javier Terol Manuel Talón Montserrat Robles Blast2GO: A universal tool for annotation, visualization and analysis in functional genomics research Bioinformatics 2005 21 3674 3676 10.1093/bioinformatics/bti610 16081474

[B24] Stephen Large-scale appearance of ultraconserved elements in tetrapod genomes and slowdown of the molecular clock Mol Biol Evol 2008 25 2 402 8 10.1093/molbev/msm268 18056681

[B25] Saccone Molecular clock and gene function Journal of molecular evolution 2003 57 Suppl 1 S277 85 10.1007/s00239-003-0037-9 15008425

[B26] Novichkov Genome-wide molecular clock and horizontal gene transfer in bacterial evolution J Bacteriol 2004 186 19 6575 85 10.1128/JB.186.19.6575-6585.2004 15375139 PMC516599

[B27] Yamamoto M Normal embryonic stages of the pygmy cuttlefish, Idiosepius pygmaeus paradoxus Ortmann Zool 1988 5 989 998 P, Sacchi N. Anal Biochem 1987, 162:156-159

