# Supplementary material for: In vitro homology search array comprehensively reveals highly conserved genes and their functional characteristics in non-sequenced species
Source: BMC Genomics. 2010 Dec 2;11(Suppl 4):S9. doi: 10.1186/1471-2164-11-S4-S9 (PMC3005928; doi:10.1186/1471-2164-11-S4-S9)
Supplement: Additional file 1 — These 67 new homologs were selected because they are detected with high intensities (>1000) in the array. [file 1471-2164-11-S4-S9-S1.pdf]

Newly identified homolg in squid head		
ProbeName	Annotation	Signal intensity
A_32_P86028_261	ribosomal protein S13 (RPS13)	6.23E+02
A_23_P150407_2458	cAMP responsive element binding protein 3-like 1 (CREB3L1)	2.11E+02
A_24_P292470_1094	uncoupling protein 3(UCP3	1.03E+02
A_23_P13048_581	keratin associated protein 5-9 (KRTAP5-9)	1.10E+02
A_23_P69497_791	C-type lectin domain family 3, member B (CLEC3B)	1.22E+02
A_23_P161595_2543	MAP/microtubule affinity-regulating kinase 2 (MARK2), transcript variant 4	1.88E+02
A_23_P366812_1181	aquaporin 5 (AQP5)	1.20E+02
A_23_P128337_898	parathymosin (PTMS)	1.90E+03
A_23_P13502_627	coiled-coil-helix-coiled-coil-helix domain containing 8 (CHCHD8)	1.96E+02
A_32_P86150_825	chymotrypsinogen B2 (CTRB2)	1.15E+02
A_23_P215491_74	chemokine (C-C motif) ligand 24 (CCL24)	2.51E+02
A_23_P38876_3654	lipase, hormone-sensitive (LIPE)	1.06E+02
A_23_P82088_1807	neuritin 1 (NRN1)	2.82E+02
A_23_P26468_607	rhomboid, veinlet-like 1 (Drosophila) (RHBDL1)	3.16E+02
A_23_P47282_2858	suppression of tumorigenicity 14 (colon carcinoma) (ST14)	1.60E+02
A_23_P64799_1530	achalasia, adrenocortical insufficiency, alacrimia (Allgrove, triple-A) (AAAS)	2.04E+02
A_23_P414884_2134	corticotropin releasing hormone receptor 1 (CRHR1), transcript variant 4	2.32E+02
A_23_P119593_1670	epoxide hydrolase 3 (EPHX3), transcript variant 2	1.41E+02
A_32_P222450_609	transmembrane protein 158 (TMEM158)	2.34E+02
A_23_P26294_1017	tryptase gamma 1 (TPSG1)	3.38E+03
A_23_P132793_787	arginine-rich, mutated in early stage tumors (ARMET)	2.55E+02
A_23_P1962_662	retinoic acid receptor responder (tazarotene induced) 3 (RARRES3)	4.84E+02
A_23_P129413_1664	dipeptidase 3 (DPEP3), transcript variant 2	1.07E+02
A_23_P126089_572	small proline-rich protein 2C (pseudogene) (SPRR2C) on chromosome 1	2.95E+02
A_23_P22382_3214	TBC1 domain family, member 10B (TBC1D10B)	4.90E+02
A_24_P279704_2299	myelin associated glycoprotein (MAG), transcript variant 1	1.14E+02
A_23_P90339_1428	splicing factor 3a, subunit 2, 66kDa (SF3A2)	3.34E+02
A_23_P76901_4306	pleckstrin homology domain containing, family G (with RhoGef domain) member 3 (PLEKHG3)	1.71E+02
A_23_P129169_1424	cytochrome P450, family 11, subfamily A, polypeptide 1 (CYP11A1), transcript variant 2	8.13E+02
A_23_P49708_2201	granulin (GRN)	4.17E+02
A_23_P163496_927	mitochondrial ribosomal protein S34 (MRPS34), nuclear gene encoding mitochondrial protein	2.22E+02
A_23_P121945_941	synuclein, beta (SNCB), transcript variant 1	1.32E+02
A_23_P116902_1260	ADP-ribosyltransferase 4 (Dombrock blood group) (ART4)	1.80E+02
A_23_P107735_1175	CD79a molecule, immunoglobulin-associated alpha (CD79A), transcript variant 2	1.78E+02
A_24_P253723_529	chromosome 17 open reading frame 91 (C17orf91), transcript variant 2	1.23E+02
A_23_P134433_3306	engrailed homeobox 2 (EN2)	2.89E+02
A_23_P28707_2281	opioid growth factor receptor (OGFR)	5.07E+02
A_24_P190190_3263	calcium channel, voltage-dependent, beta 1 subunit (CACNB1), transcript variant 1	1.91E+02
A_23_P253723_1285	retinal pigment epithelium-derived rhodopsin homolog (RRH)	1.32E+02
A_24_P10890_1109	proline rich 5 (renal) (PRR5), transcript variant 4	1.36E+02
A_24_P233078_893	peptide YY, 2 (seminalplasmin) (PYY2), non-coding RNA	5.00E+02
A_23_P132294_2786	golgi associated, gamma adaptin ear containing, ARF binding protein 1 (GGA1), transcript variant 1	1.13E+02
A_24_P216165_378	CCAAT/enhancer binding protein (C/EBP), alpha (CEBPA)	4.34E+02
A_23_P22224_694	eukaryotic translation initiation factor 4E binding protein 1 (EIF4EBP1)	3.61E+02
A_23_P5415_1270	NIF3 NGG1 interacting factor 3-like 1 (S. pombe) (NIF3L1), transcript variant 4	1.75E+02
A_24_P186943_1771	elastin (ELN), transcript variant 5	1.44E+02
A_23_P259955_1326	growth differentiation factor 5 (GDF5)	4.02E+02
A_23_P3584_2075	phospholipase A2, group XV (PLA2G15)	1.05E+02
A_24_P19054_2856	CDC42 effector protein (Rho GTPase binding) 4 (CDC42EP4)	7.98E+02
A_24_P104512_6447	envoplakin (EVPL)	1.24E+02
A_23_P391926_7589	latrophilin 1 (LPHN1), transcript variant 1	2.04E+02
A_23_P50799_867	olfactory receptor, family 10, subfamily H, member 2 (OR10H2)	6.20E+02
A_23_P108415_1524	aspartyl-tRNA synthetase (DARS)	2.20E+02
A_24_P295999_3037	CD4 molecule (CD4)	7.09E+02
A_23_P11071_1775	porcupine homolog (Drosophila) (PORCN), transcript variant E	3.83E+02
A_23_P33723_3661	CD163 molecule (CD163), transcript variant 2	1.13E+02
A_23_P126825_2585	solute carrier family 16, member 1 (monocarboxylic acid transporter 1) (SLC16A1)	1.03E+02
A_23_P168788_2748	procollagen-lysine, 2-oxoglutarate 5-dioxygenase 3 (PLOD3)	7.03E+02
A_23_P118086_2042	spermatogenesis associated 2-like (SPATA2L)	2.45E+02
A_23_P203215_924	chemokine (C-X-C motif) receptor 5 (CXCR5), transcript variant 1	1.01E+02
A_23_P218505_296	luteinizing hormone beta polypeptide (LHB)	2.58E+02
A_23_P113204_1276	fibroblast growth factor 3 (murine mammary tumor virus integration site (v-int-2) oncogene homolog) (FGF3)	1.43E+02
A_23_P142835_3462	dynactin 1 (p150, glued homolog, Drosophila) (DCTN1), transcript variant 2	1.21E+02
A_24_P209389_1448	MLX interacting protein-like (MLXIPL), transcript variant 4	3.52E+02
A_23_P150931_1970	limb region 1 homolog (mouse)-like (LMBR1L)	2.26E+03
A_24_P31583_1276	LOC729991-MEF2B readthrough transcript (LOC729991-MEF2B), transcript variant 1	4.62E+02
A_23_P207842_2685	retinoic acid receptor, alpha (RARA), transcript variant 4	1.35E+02
