# Supplementary material for: In vitro homology search array comprehensively reveals highly conserved genes and their functional characteristics in non-sequenced species
Source: BMC Genomics. 2010 Dec 2;11(Suppl 4):S9. doi: 10.1186/1471-2164-11-S4-S9 (PMC3005928; doi:10.1186/1471-2164-11-S4-S9)
Supplement: Additional file 2 — These 51 new homologs were selected because they are detected with high intensities (>1000) in the array. [file 1471-2164-11-S4-S9-S2.pdf]

Newly identified homolg in squid body		
**ProbeName**	**subjectID**	**Signal Identity**
A_23_P161792_1414	ALX homeobox 4 (ALX4)	3.24E+03
A_23_P150407_2458	cAMP responsive element binding protein 3-like 1 (CREB3L1)	1.17E+03
A_24_P292470_1094	uncoupling protein 3 (mitochondrial, proton carrier) (UCP3)	1.14E+03
A_23_P13048_581	keratin associated protein 5-9 (KRTAP5-9)	1.48E+03
A_23_P366812_1181	aquaporin 5 (AQP5)	1.56E+03
A_23_P128337_898	parathymosin (PTMS)	5.62E+03
A_23_P13502_627	coiled-coil-helix-coiled-coil-helix domain containing 8 (CHCHD8)	1.50E+03
A_32_P86150_825	chymotrypsinogen B2 (CTRB2)	1.39E+03
A_23_P215491_74	chemokine (C-C motif) ligand 24 (CCL24)	1.13E+03
A_23_P26468_607	rhomboid, veinlet-like 1 (Drosophila) (RHBDL1)	3.19E+03
A_23_P47282_2858	suppression of tumorigenicity 14 (colon carcinoma) (ST14)	1.13E+03
A_23_P414884_2134	corticotropin releasing hormone receptor 1 (CRHR1), transcript variant 4	1.92E+03
A_23_P119593_1670	epoxide hydrolase 3 (EPHX3), transcript variant 2	1.40E+03
A_32_P222450_609	transmembrane protein 158 (TMEM158)	7.19E+03
A_23_P26294_1017	tryptase gamma 1 (TPSG1)	1.85E+04
A_23_P145841_923	sclerostin domain containing 1 (SOSTDC1)	1.81E+03
A_23_P160618_1398	SH2 domain protein 2A (SH2D2A)	1.14E+03
A_23_P129413_1664	dipeptidase 3 (DPEP3), transcript variant 2	1.02E+03
A_23_P22382_3214	TBC1 domain family, member 10B (TBC1D10B)	3.21E+03
A_23_P363_1528	chromosome 1 open reading frame 113 (C1orf113)	3.21E+03
A_23_P90339_1428	splicing factor 3a, subunit 2, 66kDa (SF3A2)	2.76E+03
A_23_P64238_785	uroplakin 2 (UPK2)	1.98E+03
A_23_P76901_4306	pleckstrin homology domain containing, family G (with RhoGef domain) member 3 (PLEKHG3)	2.15E+03
A_23_P49708_2201	granulin (GRN)	3.11E+03
A_32_P86028_261	ribosomal protein S13 (RPS13)	1.30E+04
A_23_P206830_294	mitochondria-associated protein involved in granulocyte-macrophage colony-stimulating factor signal transduction	2.36E+03
A_23_P107735_1175	CD79a molecule, immunoglobulin-associated alpha (CD79A), transcript variant 2	1.56E+03
A_23_P134433_3306	engrailed homeobox 2 (EN2)	2.93E+03
A_23_P28707_2281	opioid growth factor receptor (OGFR)	3.26E+03
A_24_P10890_1109	proline rich 5 (renal) (PRR5), transcript variant 4	1.15E+03
A_24_P233078_893	peptide YY, 2 (seminalplasmin) (PYY2), non-coding RNA	6.34E+03
A_24_P216165_378	CCAAT/enhancer binding protein (C/EBP), alpha (CEBPA)	1.58E+04
A_24_P24244_1806	atrophin 1 (ATN1), transcript variant 1	1.15E+03
A_24_P186943_1771	elastin (ELN), transcript variant 5	1.57E+03
A_23_P259955_1326	growth differentiation factor 5 (GDF5)	8.67E+03
A_24_P104512_6447	envoplakin (EVPL)	3.48E+03
A_23_P63038_2445	leucine proline-enriched proteoglycan (leprecan) 1 (LEPRE1), transcript variant 1	1.16E+03
A_23_P50799_867	olfactory receptor, family 10, subfamily H, member 2 (OR10H2)	8.99E+03
A_23_P108415_1524	aspartyl-tRNA synthetase (DARS)	5.24E+03
A_24_P295999_3037	CD4 molecule (CD4)	4.03E+03
A_23_P47616_2403	folate hydrolase (prostate-specific membrane antigen) 1 (FOLH1), transcript variant 1	1.44E+04
A_23_P501831_2865	chromosome 5 open reading frame 4 (C5orf4), transcript variant 2	1.09E+03
A_23_P118086_2042	spermatogenesis associated 2-like (SPATA2L)	1.96E+03
A_23_P29773_3222	lysosomal-associated membrane protein 3 (LAMP3)	2.04E+03
A_23_P218505_296	luteinizing hormone beta polypeptide (LHB)	3.57E+03
A_23_P113204_1276	fibroblast growth factor 3(FGF3)	1.49E+03
A_24_P208513_1026	wingless-type MMTV integration site family, member 6 (WNT6)	1.28E+03
A_24_P209389_1448	MLX interacting protein-like (MLXIPL), transcript variant 4	2.65E+03
A_23_P164773_535	Fc fragment of IgE, low affinity II, receptor for (CD23) (FCER2)	1.90E+03
A_23_P15414_3190	scavenger receptor class F, member 1 (SCARF1), transcript variant 3	3.70E+03
A_23_P150931_1970	limb region 1 homolog (mouse)-like (LMBR1L)	3.41E+04
